# Radiation-Induced Toxicity Risks in Photon Versus Proton Therapy for Synchronous Bilateral Breast Cancer

**DOI:** 10.14338/IJPT-21-00023.1

**Published:** 2021-11-11

**Authors:** Line Bjerregaard Stick, Maria Fuglsang Jensen, Søren M. Bentzen, Claus Kamby, Anni Young Lundgaard, Maja Vestmø Maraldo, Birgitte Vrou Offersen, Jen Yu, Ivan Richter Vogelius

**Affiliations:** 1 Department of Oncology, Rigshospitalet, University of Copenhagen, Copenhagen, Denmark; 2 Niels Bohr Institute, Faculty of Science, University of Copenhagen, Copenhagen, Denmark; 3 Danish Center for Particle Therapy, Aarhus University Hospital, Aarhus, Denmark; 4 Greenebaum Comprehensive Cancer Center and Department of Epidemiology and Public, Health, University of Maryland School of Medicine, Baltimore, MD, USA; 5 Department of Experimental Clinical Oncology & Department of Oncology, Aarhus University Hospital, Aarhus, Denmark; 6 Department of Radiation Oncology, Miami Cancer Institute, Baptist Health South Florida, Miami, FL, USA; 7 Faculty of Health and Medical Sciences, University of Copenhagen, Copenhagen, Denmark

**Keywords:** proton therapy, bilateral breast cancer, treatment planning, bioeffect modeling, normal tissue complication probability

## Abstract

**Purpose:**

This study compares photon and proton therapy plans for patients with synchronous bilateral early breast cancer and estimates risks of early and late radiation-induced toxicities.

**Materials and Methods:**

Twenty-four patients with synchronous bilateral early breast cancer receiving adjuvant radiation therapy using photons, 3-dimensional conformal radiation therapy or volumetric modulated arc therapy, were included and competing pencil beam scanning proton therapy plans were created. Risks of dermatitis, pneumonitis, acute esophageal toxicity, lung and breast fibrosis, hypothyroidism, secondary lung and esophageal cancer and coronary artery events were estimated using published dose-response relationships and normal tissue complication probability (NTCP) models.

**Results:**

The primary clinical target volume V95% and/or nodal clinical target volume V90% were less than 95% in 17 photon therapy plans and none of the proton plans. Median NTCP of radiation dermatitis ≥ grade 2 was 18.3% (range, 5.4-41.7) with photon therapy and 58.4% (range, 31.4-69.7) with proton therapy. Median excess absolute risk (EAR) of secondary lung cancer at age 80 for current and former smokers was 4.8% (range, 0.0-17.0) using photons and 2.7% (range, 0.0-13.6) using protons. Median EAR of coronary event at age 80, assuming all patients have preexisting cardiac risk factors, was 1.0% (range, 0.0-5.6) with photons and 0.2% (range, 0.0-1.3) with protons.

**Conclusion:**

Proton therapy plans improved target coverage and reduced risk of coronary artery event and secondary lung cancer while increasing the risk of radiation dermatitis.

## Introduction

Adjuvant breast cancer radiation therapy lowers the risk of recurrence and improves survival in selected patients [1, 2]; however, it comes with a spectrum of side effects ranging from relatively mild and transient to progressive and potentially fatal conditions. Most breast cancer patients treated with radiation therapy using state-of-the-art photon therapy receive adequate target coverage with low exposure of normal tissue [3]. However, a subset of patients may benefit from favorable dose distributions that can be achieved with spot-scanning proton therapy [3–7], particularly patients with synchronous bilateral breast cancer [8, 9].

Synchronous bilateral early breast cancers are estimated to account for 1%-3% of all breast cancer incidences [10–12]. Radiation therapy planning for synchronous bilateral breast cancer is challenging, particularly in bilateral high-risk breast cancer with an indication for internal mammary node (IMN) irradiation on both sides. In 3-dimensional (3D) conformal radiotherapy (CRT), medial target coverage may be compromised to avoid tangential field overlap. Volumetric modulated arc therapy (VMAT) can improve target coverage but may result in increased exposure of heart and lungs [13] owing to a sometimes substantial low-dose bath. Comprehensive assessment of a wide range of potential adverse effects of radiation is required to provide a better picture of advantages and disadvantages of competing treatment strategies.

The aim of this study is to compare dosimetric differences in photon and proton therapy plans and estimate risks of a wide range of radiation-induced toxicity in a series of consecutive patients with bilateral breast cancer.

## Materials and Methods

Thirty-five consecutive patients with synchronous bilateral early breast cancer received photon radiation therapy at Department of Oncology, Rigshospitalet, between 2013 and 2016. Eleven patients with implants, treated with partial breast irradiation or extended clinical target volume (CTV) modifications were excluded from the study resulting in 24 included patients. The study was approved by the Danish Patient Safety Authority.

A bolus of 5 mm thickness was applied along the mastectomy scar on chest wall targets according to the guidelines of the Danish Breast Cancer Group (DBCG). The patients were CT scanned and treated in the supine position with arms above the head in either free breathing (patients receiving breast only irradiation) or using the noninvasive voluntary deep inspiration breath-hold (DIBH) technique (patients receiving lymph node irradiation) according to institutional guidelines [14]. The same CT scan was used for both the clinically delivered photon therapy plan and the comparative proton therapy plan. If patients were replanned during the course, the plan used for most fractions was selected for the treatment plan comparison.

CTVs were contoured according to DBCG guidelines [15] for patients receiving radiation therapy before 2016 and according to the European Society for Radiotherapy and Oncology (ESTRO) consensus guidelines for patients treated in 2016 [16]. Lymph nodes were for some cases irradiated and included lymph node levels II to IV, interpectoral nodes, and IMN. For some patients who had mastectomies, the primary CTV (CTVp) did include parts of the bolus. For this study, CTVp was cropped 5 mm from the body outline. Also, the heart was contoured or recontoured retrospectively following the contouring atlas by Feng et al [17]. The thyroid gland and esophagus (from the 6th cervical vertebrae to the gastro-esophageal junction) were contoured retrospectively in patients receiving local regional irradiation.

The delivered fractionation scheme was either 40 Gy in 15 fractions (2.67 Gy per fraction) or 50 Gy in 25 fractions (2 Gy per fraction), and it was not necessarily the same for right- and left-sided targets within the same patient. The fractionation scheme was decided according to the guidelines of DBCG and multidisciplinary team discussions. Three patients received unilateral sequential boost of 10 Gy in 5 fractions (1 patient) or 16 Gy in 8 fractions (2 patients), but the boosts were not considered for the purpose of this study.

Clinical planning objectives for patients treated before 2016 were to cover CTVp (whole breast or the chest wall) with 95% to 107% and lymph node CTVs with 90% to 107% of the prescribed dose. Clinical planning objectives for patients receiving radiation therapy in 2016 were V95% (volume that received at least 95% of the prescribed dose) ≥ 98% for CTVp, V90% ≥ 98% for lymph node CTVs, and V107% ≤ 2% for all CTVs. All comparative proton plans were created following the 2016 planning objectives. Dose to normal tissues should be as low as reasonably achievable with specific dose objectives for the heart and lungs (heart: V20Gy/V17Gy ≤ 10% (50Gy in 25 fractions / 40Gy in 15 fractions) and V40Gy/V35Gy ≤ 5% for local regional irradiation and V17Gy ≤ 5% and V35Gy ≤ 1% for breast only irradiation; each lung: V20Gy/V17Gy ≤ 35% and mean dose ≤ 18Gy/16Gy for local regional irradiation and V17Gy ≤ 25% and mean dose ≤ 16 Gy for breast only irradiation. Challenging cases were discussed in the clinic, and for some patients the dose objectives were compromised.

### Photon Therapy Planning

**Figure 1.**
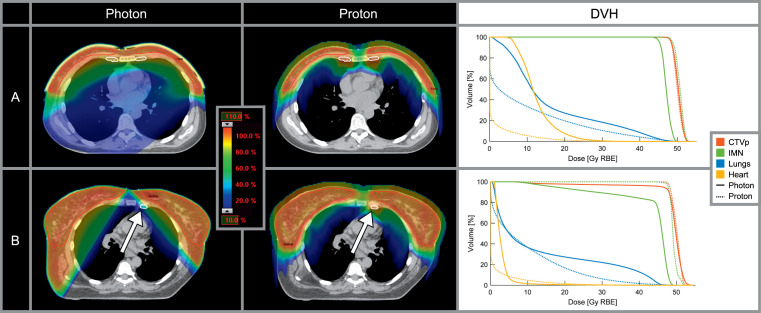
Examples of dose distributions for the clinically delivered photon therapy plan and the comparative proton therapy plan for 2 patients treated with 50 Gy in 25 fractions. The color wash shows 10% to 110% of the prescribed dose. For patient A, adequate coverage of CTVp and IMN was achieved with VMAT but resulted in high exposure of the heart. For patient B (treated with 3D CRT), coverage of CTVp and IMN was compromised (white arrow) to avoid overlapping fields. Adequate target coverage and low exposure of the heart were achievable with protons for both patients. Abbreviations: CRT, conformal radiotherapy; CTVp, primary clinical target volume (whole breast or chest wall); DVH, dose-volume histogram; IMN, internal mammary nodes; RBE, relative biological effectiveness; VMAT, volumetric arc therapy.

The clinically delivered photon therapy plans were created using the 3D CRT or VMAT technique (see **Figure 1**). Three-dimensional CRT for patients with no lymph node targets consisted of opposing tangential fields using 2 isocenters centrally placed in the left and right breasts. For patients receiving local regional irradiation, periclavicular fields were added with a field junction at the isocenter. Fields used 6 MV photons as well as field-in-field techniques with supplementary 18 MV fields to ensure homogeneous target coverage. Planning target volumes were not used with 3D CRT, instead the multileaf collimator was adapted to the CTV with a margin of 1 cm for each field. The multileaf collimators were subsequently modified in some patients to shield the heart.

VMAT plans were created with a single isocenter posterior to the sternum and comprised 2 to 3 partial arcs ranging between 220° and 140°. Planning target volumes were generated with a 5 to 7 mm margin to the CTV and afterwards contracted to 3 to 5 mm under the skin surface.

The photon therapy plans were generated in the Eclipse treatment planning system (Varian Medical Systems, Palo Alto, California) and calculated using the analytical anisotropic algorithm or the AcurosXB algorithm. For this study, all photon therapy plans were recalculated with the AcurosXB algorithm.

### Proton Therapy Planning

The pencil beam scanning proton therapy plans were created in the Eclipse treatment planning system using multifield optimization (see **Figure 1**). Relative biological effectiveness (RBE) was assumed to be 1.1. Two isocenters were used; 1 centrally aligned with the right-sided CTVs and 1 centrally aligned with the left-sided CTVs. Four to 6 *en face* fields were used with 2 to 3 per isocenter. Field angles for right-sided targets ranged from 310° to 10° and field angles for left-sided targets ranged from 350° to 50°. Medial hot spots in patients receiving local regional irradiation could be reduced using anterior-posterior field directions (350°-10°) for some of the fields. A range shifter of water-equivalent thickness of 57 mm was applied to cover the superficial target areas. The distance between isocenter and snout position was set to 25 cm. Spots were placed in a hexagonal grid with spot spacing of 0.350 to 0.425 times the full width at half maximum of the spot in air at the given energy. Bolus and superficial clips and wires were delineated, and the density of these structures was assigned to be air by overriding the Hounsfield units.

The CTVs and heart objectives were optimized robustly using 14 plan uncertainty parameters: ±0.5 cm isocenter shift in x, y, and z direction ±3.5% range uncertainty (12 scenarios) and ±3.5% range uncertainty (2 scenarios). Field uncertainty parameters of ±0.5 cm in lateral direction were applied to account for uncertainty of right- and left-sided fields overlapping when using 2 isocenters. Optimisation structures were created by expanding the CTVs with a 5 mm margin to help increase the dose at the border of the CTV to achieve robust target coverage. The plans were optimized with the nonlinear universal proton optimizer (NUPO) algorithm, and dose calculations were carried out using the proton convolution superposition algorithm with a 0.25-cm dose grid. The plans were normalized such that mean dose to the combined CTVs equaled prescription dose.

The plan creation process had additional steps if different fractionation schemes were used for the right- and left-sided targets: the plan was created and optimized as previously described using 50 GyRBE in 25 fractions; the plan was then copied, and right-sided fields were deleted in one of the plans and left-sided fields were deleted in the other plan. The fractionation scheme was then changed to 40 GyRBE in 15 fractions for the relevant laterality, and the 2 plans were afterwards summed. When reducing dose per fraction, spots may be deleted owing to a minimum allowed spot weight, and thus it was important to go from a 2-GyRBE dose per fraction to 2.67 GyRBE.

### Plan Evaluation

Homogeneity index (HI) was calculated for the CTVp and defined [18] as:



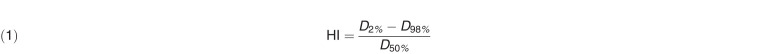

D2%, D50%, and D98% represent the dose received by 2%, 50%, and 98% of the CTVp, respectively.

Plan uncertainty evaluation against uncertainty in patient positioning in ± x, y, and z (0.5 cm) ± proton beam range (3.5%) were estimated using integrated evaluation tool in Eclipse for all proton plans (14 scenarios). The objective for the plan uncertainty was that at least 13 out of 14 scenarios should fulfil V95% ≥ 95% for CTVp and V90% ≥ 95% for the individual lymph nodes.

Paired, 2-tailed Wilcoxon signed rank test was used to compare dose metrics and estimated risks in the photon versus proton therapy plans.

### Bioeffect Modeling

We aimed to provide a comprehensive assessment of the risk of toxicity and second malignancy induction following the competing treatment plans. See **Table 1** for a summary of the bioeffect modeling studies underlying the applied models and **Supplemental Appendix A** for a detailed description of the models. We prioritized models based on breast cancer; however, in some cases such data were not available, and we reverted to using data based on non–small cell lung cancer.

**Table 1.** Summary of normal tissue complication models used in the present work.

**Endpoint**	**Study design**	**Population**	**Model type**	**Model variables**	**α/β ratio**	**Model references**
Early toxicities						
Radiation dermatitis grade ≥2	Cohort series	Breast cancer	Logistic	EQD2 max to skin, radiation type	10 [19]	DeCesaris et al 2019 [19]
Radiation pneumonitis grade ≥1	Pooled multi-center study	Thoracic cancers (mostly lung cancer)	Logistic	Mean lung dose^a^	4.0 [20]	Marks et al 2010 [21]
Acute esophagus toxicity grade ≥2	Cohort series	Non–small cell lung cancer	Logistic	Esophagus V35Gy	NA	Belderbos et al 2005 [22]
Late, non–life-threatening						
Lung fibrosis grade ≥2	Cohort series	Non–small cell lung cancer	Lyman	EUD for lungs	3.1 [23]	Tucker et al 2018 [24]
Breast fibrosis ≥ moderate	Pooled multi-center RCT study	Breast cancer	Logistic	BEUD for breast	3 [25]	Mukesh et al 2013 [25]
Hypothyroidism	Cohort series	Breast cancer	Logistic	Thyroid gland volume receiving less than 20 Gy	NA	Huang et al 2021 [26]
Late, life-threatening						
Coronary artery events at age 80	Population-based case- control study	Breast cancer	Linear no- threshold	Mean heart dose,^a^ age, cardiac risk factors	2 [27]	Darby et al 2013 [27]
Secondary lung cancer at age 80	Nested case-control study	Breast cancer	Linear no- threshold	Mean lung dose, age, smoking status	NA	Grantzau et al 2014 [28]^b^
Secondary esophageal cancer at age 80	Nested case-control study	Breast cancer	Linear no- threshold	Mean esophagus dose, age	NA	Journy et al 2020 [29]^c^

**Abbreviations:** NA, not applicable; EQD2, equivalent dose in 2 Gy fractions; EUD, equivalent uniform dose; BEUD, biologically effective uniform dose.

a Biological equivalent, in 25 fractions, see text for details.

b General population lung cancer incidence rates for females in Denmark from NORDCAN [30], general population survival rates for females in Denmark from Statistics Denmark [31], rates of current and former smokers in Denmark from Danish Health Authority [32], percentage of smoking-related lung cancer deaths from Centers for Disease Control and Prevention (CDC) [33].

c General population esophageal cancer incidence rates for females in Denmark from NORDCAN [30], general population survival rates for females in Denmark from Statistics Denmark [31].

The toxicities were grouped in 3 overall categories (a-c):

- Early toxicities
  - Radiation dermatitis grade ≥ 2 [19]
  - Radiation pneumonitis grade ≥ 1 [21]
  - Acute esophageal toxicity grade ≥ 2 [22]
- Late, non–life-threatening toxicities:
  - Lung fibrosis grade ≥ 2 [24]
  - Breast fibrosis grade ≥ moderate [25]
  - Hypothyroidism [26]
- Late, life-threatening toxicities:
  - Coronary artery event at age 80 [27]
  - Secondary lung cancer at age 80 (for current or former smokers) [28]
  - Secondary esophageal cancer at age 80 [29]

For patients treated with 2 fractionation schemes, the 40 Gy in 15 fractions were converted to 50 Gy in 25 fractions for the purpose of modeling risks. For patients treated in 15 fractions, the dose metric used in the models for radiation pneumonitis and coronary artery events were converted to the corresponding biological equivalent dose metric in 25 fractions (**Equation A2** in Supplemental Appendix A). Relevant α/β ratios are provided in **Table 1**. Acute esophageal toxicity, hypothyroidism, and secondary esophageal cancers were only modeled for patients receiving local regional irradiation.

Late, life-threatening toxicities were modeled as excess absolute risk (EAR) of having an event before the age of 80 years. Secondary lung and esophageal cancers were modeled following the methodology in Brodin et al [34]. The EAR of secondary lung cancer for never-smokers was assumed to be 0% since Grantzau et al [28] found a non–statistically significant excess relative risk of 0.06 per Gy to the center of the lung tumor for never-smokers.

Coronary artery events were defined as myocardial infarction, coronary revascularization, death from ischemic or unstable angina [27]. The EAR of a coronary artery event was modeled twice for each patient: first assuming no preexisting cardiac risk factors and second assuming preexisting cardiac risk factors. Cardiac risk factors included current smoking, body mass index ≥ 30, and a history of ischemic heart disease, diabetes, or chronic obstructive pulmonary disease [27].

## Results

Median patient age was 65 years (range, 33-81). Patient and treatment characteristics are provided in **Table 2**.

**Table 2.** Patient and treatment characteristics for the 24 patients included in the study.

**Parameter**	**Value, n (%)**	
	**Left side**	**Right side**
Tumor histology		
Invasive ductal carcinoma	15 (63)	16 (67)
Invasive lobular carcinoma	6 (25)	5 (21)
Ductal carcinoma in situ	3 (13)	3 (13)
Tumor size		
T1 (<20 mm)	11 (46)	12 (50)
T2 (20–50 mm)	8 (33)	7 (29)
T3 (>50 mm)	2 (8)	2 (8)
Not applicable^a^	3 (13)	3 (13)
Malignancy grade, ductal only		
I	4 (17)	4 (17)
II	15 (63)	13 (54)
III	2 (8)	3 (13)
Not applicable^a^	3 (13)	3 (13)
Unknown	0 (0)	1 (4)
Number of positive LN		
0	9 (38)	15 (63)
1–3	9 (38)	6 (25)
4–9	2 (8)	0 (0)
≥10	1 (4)	0 (0)
Not applicable^a^	3 (13)	3 (13)
HER2 status		
Positive	2 (8)	0 (0)
Negative	19 (79)	21 (88)
Not applicable^a^	3 (13)	3 (13)
ER status		
Positive	19 (79)	20 (83)
Negative	2 (8)	1 (4)
Not applicable^a^	3 (13)	3 (13)
	**value (%)**	
Adjuvant chemotherapy		
Epirubicin+Cyclophosphamide+Docetaxel	8 (33)	
Epirubicin+Cyclophosphamide+Paclitaxel	3 (13)	
None	12 (50)	
Unknown	1 (4)	
Dose-fractionation scheme		
40 Gy in 15 fractions	11 (46)	
50 Gy in 25 fractions	9 (38)	
Combination	4 (17)	
CTVp		
Bilateral chest wall	4 (17)	
Bilateral whole breast	16 (67)	
Chest wall & whole breast	4 (17)	
Lymph node irradiation		
No LN irradiation	12 (50)	
Left side only	6 (25)	
Right side only	1 (4)	
Both sides	5 (21)	
Photon therapy planning technique		
3D CRT	21 (88)	
VMAT	3 (13)	
Free breathing or DIBH		
Free breathing	12 (50)	
DIBH	12 (50)	

**Abbreviations:** n, number of patients; ER, estrogen receptor; HER2, human epidermal growth factor receptor 2; CTVp, primary clinical target volume; LN, lymph node; CRT, conformal radiotherapy; VMAT, volumetric arc therapy; DIBH, deep inspiration breath-hold.

a Ductal carcinoma in situ.

The CTVp V95% was less than 95% in 12 photon therapy plans (6 plans including lymph node irradiation) and less than 90% in 3 photon plans (1 plan including lymph node irradiation) for either left, right or both CTVp. The nodal CTV (CTVn) V90% was less than 95% in 10 photon plans and less than 90% for 7 photon plans for at least one of the CTVn. The CTVp V95% and the CTVn V90% in the proton therapy plans were 97.8% or more. The median HI for CTVp was 0.10 (range, 0.07-0.95) using photons and 0.08 (range, 0.06–0.11) using protons (*P* < .001).

**Figure 2.**
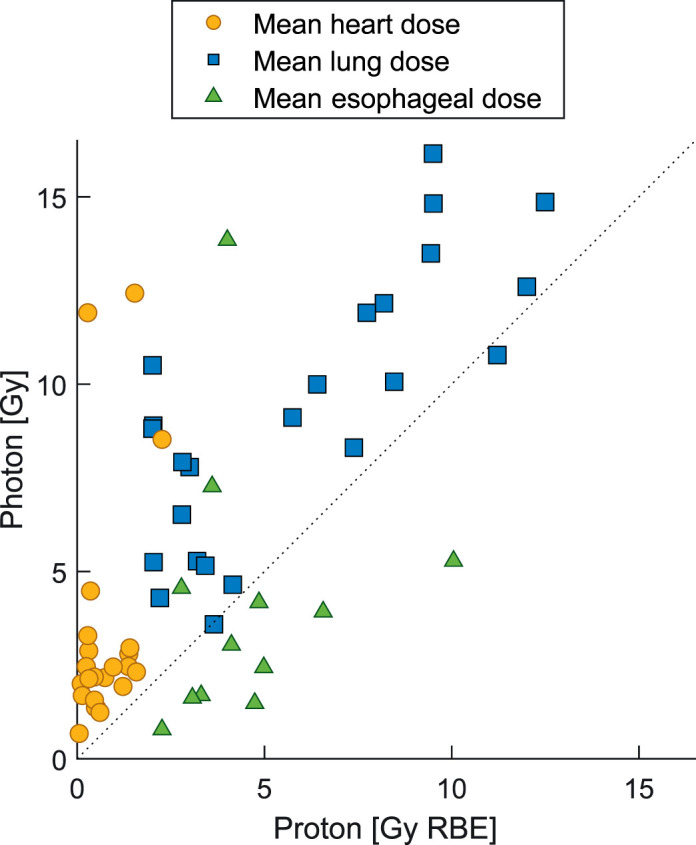
Mean doses to heart, whole lung, and esophagus from the photon and competing proton therapy plan for each patient. Esophagus and thyroid gland were only delineated and evaluated in patients receiving lymph node irradiation (12 patients). The mean doses in this figure were not fractionation corrected.

Median mean heart dose was 2.3 Gy (range, 0.7-12.4) for photon therapy plans versus 0.5 Gy RBE (range, 0.1-2.3) for proton therapy plans (*P* < .001), see **Figure 2**. Median V20Gy for both lungs was 18.9% (range, 5.7-30.4) using photons and 7.1% (range, 0.8-28.6) using protons (*P* < .001). Dosimetric results are provided in **Table 3**.

**Table 3.** Dosimetric results for the clinically delivered photon therapy plan and the competing proton therapy plan. Dose metrics in this table were reported assuming RBE = 1.1, and the metrics were not fractionation corrected. Esophagus and thyroid gland were only delineated and evaluated in patients receiving local regional irradiation (12 patients). Note that patients receiving breast only irradiation were treated in free breathing, and patients receiving local regional irradiation were treated in deep inspiration breath-hold. Abbreviations: CTVp, primary clinical target volume; LN, lymph nodes; IMN, internal mammary nodes; HI, homogeneity index; VXX%, volume that received at least XX% of the prescribed dose; VXXGy, volume that received at least XX Gy; CV20Gy, volume that received less than 20 Gy.

	**No LN irradiation (12 patients)**		**LN irradiation (12 patients)**	
	**Photon, median (range)**	**Proton, median (range)**	**Photon, median (range)**	**Proton, median (range)**
CTVp				
Left CTVp V95%, %	96.4 (83.5–99.2)	99.5 (98.0–100)	98.2 (91.3–100)	98.9 (97.8–99.9)
Right CTVp V95%, %	98.0 (90.2–99.2)	99.4 (98.3–100)	95.7 (87.5–100)	99.1 (98.0–99.9)
HI CTVp	0.09 (0.07–0.95)	0.08 (0.06–0.09)	0.12 (0.07–0.89)	0.09 (0.07–0.11)
LN, %				
Left LN V90%	–	–	97.0 (87.9–100)	99.7 (99.1–100)
Right LN V90%	–	–	91.0 (81.4–99.8)	99.8 (99.3–100)
IMN, %				
Left IMN V90%	–	–	82.2 (0.7–100)	99.9 (98.6–100)
Right IMN V90%	–	–	15.3 (0.6–98.2)	100 (98.3–100)
Heart				
Mean dose, Gy	2.1 (0.7–11.9)	0.3 (0.1–0.5)	2.4 (1.2–12.4)	1.3 (0.5–2.3)
V20Gy, %	1.7 (0.0–5.5)	0.0 (0.0–0.3)	0.5 (0.0–7.7)	1.1 (0.0–2.2)
Lungs				
Mean dose, Gy	5.9 (3.6–10.5)	2.8 (2.0–4.2)	12.0 (8.3–16.2)	9.0 (5.7–12.5)
V20Gy, %	10.2 (5.7–20.1)	2.2 (0.8–6.3)	23.3 (16.3–30.4)	16.5 (7.8–28.6)
Left lung				
Mean dose, Gy	5.9 (3.2–9.8)	2.7 (1.2–4.0)	14.8 (6.2–17.7)	10.1 (3.9–14.4)
V20Gy, %	10.3 (5.5–20.6)	2.1 (0.1–6.4)	26.8 (13.1–36.5)	20.6 (5.3–30.8)
Right lung				
Mean dose, Gy	6.5 (3.4–11.0)	2.8 (2.0–4.3)	10.5 (5.6–16.3)	8.9 (4.0–12.2)
V20Gy, %	11.3 (5.1–24.5)	2.5 (1.1–6.3)	20.2 (8.8–34.1)	16.3 (3.0–26.7)
Esophagus				
Mean dose, Gy	–	–	3.5 (0.8–13.8)	4.1 (2.3–10.0)
Maximum dose, Gy	–	–	36.1 (2.9–43.8)	43.2 (25.9–49.3)
V35Gy, %	–	–	0.05 (0.0–3.5)	1.6 (0.0–7.1)
Thyroid gland				
Mean dose, Gy	–	–	20.6 (2.9–42.1)	22.3 (2.8–49.3)
Maximum dose, Gy	–	–	50.6 (33.4–52.8)	51.3 (29.8–52.6)
CV20Gy, cm^3^	–	–	5.7 (1.2–14.7)	4.2 (0.0–15.6)
Skin, %				
Maximum dose	104.8 (100.9–106.7)	103.0 (99.9–106.7)	108.0 (98.6–119.3)	102.6 (100.3–107.3)
Body, cm^3^				
V107%	0.0 (0.0–9.6)	0.1 (0.0–4.4)	15.3 (1.1–42.5)	0.1 (0.0–0.8)

**Figure 3.**
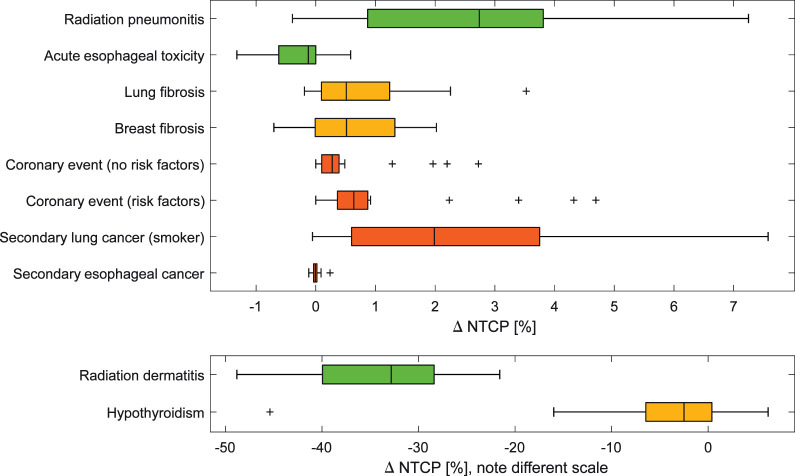
Boxplot of the delta normal tissue complication probability (NTCP) for the studied toxicities. Δ NTCP was defined as NTCP for photons minus NTCP for protons. The toxicities were categorized in early (green), late, non–life-threatening (yellow), and late, life-threatening (red). Note the different scale for delta NTCP for radiation dermatitis and hypothyroidism.

Median NTCP of radiation dermatitis ≥ grade 2 was 18.3% (range, 5.4-41.7) with photon therapy and 58.4% (range, 31.4-69.7) with proton therapy. Median EAR of coronary event at age 80 assuming all patients having preexisting cardiac risk factors was 1.0% (range, 0.0-5.6) with photons and 0.2% (range, 0.0-1.3) with protons. Median EAR of secondary lung cancer at age 80 for current and former smokers was 4.8% (range, 0.0-17.0) using photons and 2.7% (range, 0.0-13.6) using protons. Risk estimates for all the studied radiation-induced toxicities for the delivered photon therapy plans and the competing proton therapy plans are provided in **Table B1** and in **Figure B1** in **Supplemental Appendix B**. **Figure 3** shows a box plot of difference in NTCP between photon and proton therapy for the studied endpoints.

## Discussion

For patients with bilateral breast cancer, we observed compromises on CTVp coverage (V95% < 95%) for half the patients with the delivered photon plans (all treated with 3D CRT), often to avoid extensive dose to the heart or to avoid overlap between the tangential fields. Adequate target coverage could probably have been obtained using the VMAT technique, but might, in return, increase the dose to the heart and lungs.

It is difficult to weigh the importance of different early and late toxicity risks. However, studies looking at one endpoint at a time may inadvertently bias toward one of the modalities. In this study, we observed a reduced risk of coronary artery events and lung endpoints but a greater risk of radiation dermatitis and larger exposure of the esophagus with the proton therapy plans compared with the photon therapy plans.

The DBCG-IMN study found that IMN irradiation improved overall survival in patients with high-risk unilateral breast cancer [35] and the NCIC-CTG MA.20 and EORTC 22922/10925 randomized controlled trials showed that regional lymph node irradiation reduced the rate of breast cancer recurrence [36–38]. Accordingly, a subgroup of our patients for whom large compromises on target coverage were made may have a clinically relevant benefit from adequate target coverage obtained with proton therapy. It should be noted that tumor control and survival data in synchronous bilateral breast cancer are limited [39].

In the DBCG Proton trial (ClinicalTrials.gov number, NCT04291378) [40], breast cancer patients are randomized between photon radiation therapy and proton therapy. Full target coverage is a prerequisite and patients may be included if exposure to the heart or lungs exceeds a prespecified threshold with photon therapy [41]. After randomization, the optimal proton and photon plans are made (including possible target compromises as deemed optimal by treating physician). We suspect that most patients in the present study would have been candidates for the trial if treated today, especially patients referred for lymph node irradiation including the IMN. The Dutch proton therapy strategy is a model-based selection [42, 43] using delta NTCP ≥ 2% of coronary artery event at age 80 [27] as plan selection criteria. In this study, 2 patients had an estimated delta NTCP ≥ 2% assuming no cardiac risk factors, and 4 patients, assuming at least 1 risk factor. However, as illustrated by our analyses, comprehensive assessment of both target compromises and several competing endpoints are relevant for such selection processes.

The clinically delivered photon therapy plans were created in our department between 2013 and 2016; thus, recent improvements in photon therapy planning, such as knowledge-based treatment planning [44] or tangential VMAT planning [45], might allow for improved dose distributions. All patients receiving breast only irradiation were treated in free breathing, and treatment in DIBH could potentially have reduced the dose to the heart and lungs [46], and an expansion of the chest might have allowed for wider tangential angles. Proton therapy plans for breast cancer have been shown to be robust against respiratory motion when using an *en face* field arrangement [47, 48], and treatment planning studies have indicated no benefit from DIBH in proton therapy [5, 6] despite the caudal displacement of the heart in DIBH, moving it away from the IMN.

This study presents a variety of modeled radiation-induced risks, all of which come with substantial uncertainties that should be acknowledged. The models for radiation pneumonitis, acute esophageal toxicity, and lung fibrosis are based on predominately non–small cell lung cancer patients; it should be acknowledged that patients in this group are frailer, more often have comorbidities, and receive other dose distributions, concurrent chemotherapy, and fractionation schemes. Coronary artery events were modeled using dose-response data from a population-based case-control study [27] in which none of the patients received taxanes or trastuzumab and only a few were treated with anthracyclines; these treatments are known to affect the heart [49]. All the models, except for the radiation dermatitis model, are based on photon therapy data having different dose distributions and different biological effects than protons. For this study, the CTVp was cropped 5 mm from the body outline for all chest wall targets, which might have resulted in an overestimation of the maximum dose to skin (and therefore, an overestimation of the risk of radiation dermatitis) in photon therapy plans. The risks of telangiectasia, cosmesis, and brachial plexopathy were considered for the risk assessment; however, the risks were considered negligible as they depend on a dose above 108% to 110% of the prescription dose, which is restricted in DBCG guidelines [50]. We did not manage to establish models for rib fracture [51], oedema, arm, or shoulder morbidity.

A comprehensive toxicity risk assessment, using data-driven risk estimates, was conducted for 24 patients with synchronous bilateral breast cancer. Compromises in target coverage were seen in the clinical 3D CRT plans, whereas proton therapy plans could have provided sufficient target coverage, especially patients referred for lymph node irradiation including the IMN. Proton therapy led to the lowest estimated risk of radiation pneumonitis, lung and breast fibrosis, coronary artery events, and secondary lung cancer; however, it also led to a substantially higher risk of radiation dermatitis.
