# Supplementary material for: Radiation-Induced Toxicity Risks in Photon Versus Proton Therapy for Synchronous Bilateral Breast Cancer
Source: Int J Part Ther. 2021 Nov 11;8(4):1–13. doi: 10.14338/IJPT-21-00023.1 (PMC9009461; doi:10.14338/IJPT-21-00023.1)
Supplement: Supplementary file 1 [file ijpt-08-04-07_s01.pdf]

Supplementary material

Radiation-induced toxicity risks in photon vs proton therapy for synchronous bilateral breast cancer

Line Bjerregaard Stick, Maria Fuglsang Jensen, Søren M. Bentzen, Claus Kamby, Anni Young Lundgaard, Maja Vestmø Maraldo, Birgitte Vrou Offersen, Jen Yu, Ivan Richter Vogelius

Appendix A

Bioeffect modelling

Dose from proton therapy plans was in Gy RBE assuming a fixed RBE of 1.1.

Models for radiation pneumonitis and coronary events used dose metrics that were biological equivalent fractionation-corrected: the dose metrics for patients treated in 15 fractions were converted into the corresponding biological equivalent dose metric in 25 fractions as follows:

$$EQD_2 = D\frac{D/15 + \alpha/\beta}{2 + \alpha/\beta} \tag{A1}$$

$$D\frac{D/15 + \alpha/\beta}{2 + \alpha/\beta} = D'\frac{D'/25 + \alpha/\beta}{2 + \alpha/\beta} \tag{A2}$$

D = the dose metric in 15 fractions.
D' = the corresponding biological equivalent dose metric in 25 fractions.

Early toxicities

Radiation dermatitis grade ≥2

Logistic model from DeCesaris et al. 2019 (1). Cohort study on 86 breast cancer patients treated with photon therapy (3D CRT or IMRT) or pencil beam scanning proton therapy.

$$NTCP = \frac{1}{1 + exp - (0.1342 \cdot EQD_2MaxSkin - 2.150 \cdot Radiation - 4.297)} \tag{A3}$$

α/β for radiation dermatitis: 10 from DeCesaris et al. 2019 (1).
Model variables: $Radiation = 1$ for protons, $Radiation = 2$ for photons and $EQD_2MaxSkin =$ maximum EQD_2 to skin (3 mm thick) in Gy.

Radiation pneumonitis grade ≥1

Logistic model from QUANTEC (Marks et al. 2010) (2). Pooled multi-centre study on thoracic cancers (mostly non-small cell lung cancer).

$$NTCP = \frac{1}{1 + exp\left(4 \cdot \gamma_{50} \cdot \left(1 - \frac{MLD}{D_{50}}\right)\right)} \tag{A4}$$

Model parameters: $D_{50} = 30.8$ Gy (95% CI: 28.7 to 33.9) and $\gamma_{50} = 0.97$ (95% CI: 0.83 to 1.12).
α/β for radiation pneumonitis: 4.0 Gy (95% CI: 2.2 to 5.8) from Bentzen et al. 2000 (3).
Model variable: $MLD =$ Biological equivalent fractionation-corrected mean dose to whole lung in Gy.

Acute oesophageal toxicity grade ≥2

Logistic model from Belderbos et al. 2005 (4). Cohort study on 156 non-small cell lung cancer patients treated with radiotherapy or sequential chemo-radiotherapy (2.25 to 2.75 Gy per fraction).

$$NTCP = \frac{1}{1 + exp - (-3.37 + 0.05 \cdot V35_{Gy})} \tag{A5}$$

α/β for acute oesophageal toxicity: not necessary. Oesophagus were only delineated for patients receiving loco-regional irradiation, and all patients receiving loco-regional irradiation were treated with 50 Gy, thus, no need for fractionation-corrected doses.
Model variable: $V35Gy =$ percentage of the oesophagus volume that receives 35 Gy or more.

Late, non-life-threatening toxicities

Lung fibrosis grade ≥2

Lyman model from Tucker et al. 2018 (5). Cohort study on 203 non-small cell lung cancer treated in a randomised controlled trial with IMRT or passive scattering proton therapy with 66 to 74 Gy in 33 to 37 fractions and concurrent chemotherapy.

$$EUD = \left(\sum_i V_i (D_i)^{1/n} \right)^n \tag{A6}$$

$$t = \frac{EUD - TD_{50}}{m \cdot TD_{50}} \tag{A7}$$

$$NTCP = \frac{1}{\sqrt{2\pi}} \int_{-\inf}^{t} e^{-u^2/2} du \tag{A8}$$

Model parameters: $n = 0.48$ (95% CI: 0.20 to 0.76), $m = 0.21$ 1/Gy (95% CI: 0.11 to 0.31) and $TD_{50} = 35.7$ Gy (95% CI: 23.8 to 47.6).
α/β for lung fibrosis: 3.1 (95% CI: -0.2 to 8.5) from Basic Clinical Radiobiology (6).
Model: variables: V = volume of the voxel i in the whole lung, D = absolute dose in Gy for voxel i in the whole lung.

Breast fibrosis grade ≥ moderate

Logistic model from Mukesh et al. 2013 (7). Pooled multi-centre study using individual dose–volume and toxicity data from 574 breast cancer patients (50%) from the Cambridge IMRT trial and 5282 breast cancer patients (95%) from the EORTC "boost vs no boost" trial for the NTCP modelling. Breast fibrosis was evaluated at 5 years in the Cambridge trial and at 10 years in the EORTC trial.

$$EUD = \left(\sum_i V_i (D_i)^{1/n} \right)^n \tag{A9}$$

$$BEUD = EUD \left(1 + \frac{EUD/n_{fractions}}{\alpha/\beta} \right) \tag{A10}$$

$$NTCP = \left(\frac{1}{1 + \left(\frac{BEUD_{50}}{BEUD}\right)^{4\gamma_{50}}} \right) \tag{A11}$$

α/β for breast fibrosis: 3 Gy from Mukesh et al. 2013 (7).
Model parameters: $n = 0.011$ (95% CI: 0.1 to 0.3), $\gamma_{50} = 0.9$ (95% CI: 0.84 to 0.97), $BEUD_{50} = 136.4$ (95% CI: 132.8 to 140).
Model variables: V = volume of the voxel i in CTVp for both breasts, D = absolute dose in Gy for voxel i CTVp for both breasts and nfractions = number of fractions (15 or 25).

Hypothyroidism

Logistic model from Huang et al. 2021 (8). Cohort study on 192 breast cancer patients receiving radiotherapy (3D CRT or IMRT) that included supraclavicular loco-regional irradiation.

$$NTCP = \frac{1}{1 + exp - (-0.405 + 0.011 \cdot FU - 0.234 \cdot CV20_{Gy})} \tag{A12}$$

α/β for hypothyroidism: not necessary. The thyroid gland was only delineated for patients receiving loco-regional irradiation, and all patients receiving loco-regional irradiation were treated with 50 Gy, thus, no need for fractionation-corrected doses.
Model variables: $CV20Gy$ = absolute thyroid gland volume receiving less than 20 Gy in cc and FU = follow-up in months. In our study, hypothyroidism was estimated assuming $FU = 60$ months.

Late, life-threatening toxicities

Excess absolute risks (EAR) for radiation-induced secondary lung cancer and secondary oesophageal cancer at age 80 were modelled following the approach presented by Brodin et al. 2012 (9).

$$EAR_x = ERR_x \cdot D \cdot \int_{age}^{80} I_{baseline,x}(a, sex) \cdot S_{baseline,x}(a, sex)da \tag{A13}$$

EAR_x = excess absolute risk for complication x.
ERR_x = excess relative risk for complication x.
D = dose metric.
$I_{baseline,x}(a, sex)$ = general population incidence rate for complication x which depends on age (a) and sex.
$S_{baseline}(a, sex)$ = general population survival rate which depends on age (a) and sex. Values for $S_{baseline}(a, sex)$ were found from Statistics Denmark (10), see Table A1.

The integral was from patient age at treatment to 80 years. One of the patients was 81 years old and the patient's EAR of late, life-threatening toxicities was consequently 0%.

Age	Survivors per 100,000	Age	Survivors per 100,000	Age	Survivors per 100,000
20	99513	40	98920	60	94636
21	99498	41	98849	61	94119
22	99481	42	98780	62	93513
23	99465	43	98706	63	92849
24	99448	44	98620	64	92141
25	99434	45	98529	65	91424
26	99418	46	98423	66	90586
27	99395	47	98318	67	89711
28	99377	48	98180	68	88782
29	99353	49	98016	69	87800
30	99327	50	97858	70	86754
31	99301	51	97665	71	85583
32	99267	52	97454	72	84292
33	99239	53	97203	73	82903
34	99200	54	96942	74	81353
35	99168	55	96636	75	79753
36	99135	56	96293	76	77973
37	99089	57	95949	77	75995
38	99041	58	95556	78	73843
39	98981	59	95118	79	71479

Table A1: Survivors per 100,000 by age for females in Denmark in 2015-2019 (10).

Secondary lung cancer at age 80

ERR/Gy = 0.173 (95% CI: 0.045 to 0.540) for ever-smokers from the nested case-control study on breast cancer by Grantzau et al. 2014 (11). EAR of secondary lung cancer was modelled for current and former smokers using the ERR/Gy estimate for ever-smokers with 96 cases from Grantzau et al. Mean dose to the whole lung was used as dose variable (note that Grantzau et al. used dose to the centre of the second lung tumour). We assumed that second malignancy induction was independent of fractionation, and consequently no fractionation corrected doses were not used.

EAR of secondary lung cancer was assumed to be 0% for never-smokers since Grantzau et al. in nine cases (never-smoker or unknown smoking status) found a non-statistically significant ERR/Gy of 0.006 (95% CI: -0.020 to 0.163).

The incidence rate of lung cancer in the general population was found in the NORDCAN database (12), see Table A2.

Age	Incidence per 100,000	Incidence in %
20-24	0.6	0.0006%
25-29	0.0	0.0000%
30-34	1.7	0.0017%
35-39	1.9	0.0019%
40-44	2.3	0.0023%
45-49	4.6	0.0046%
50-54	20.8	0.0208%
55-59	61.5	0.0615%
60-64	103.8	0.1038%
65-69	185.3	0.1853%
70-74	257.0	0.2570%
75-79	307.8	0.3078%

Table A2: Incidence rates of lung cancer by age in the female general population (never-smokers, current smokers and former smokers) in Denmark from 2016 (12).

The incidence rates of lung cancer in general population (never smokers, current smokers and former smokers) were converted to incidence rates of lung cancer for current and former smokers only the following way: The Danish Health Authority estimated 44.5% never-smokers in Denmark in 2018 corresponding to 55.5% current or former smokers (13). 80 to 90% of all lung cancer deaths are smoking-related (14), and we assumed 85% of all lung cancer events were related to smoking.

Incidence rates in Table A2 were converted by multiplying each entry with 0.85/0.555. See Table A3 for incidence rates of lung cancer in the smoking (current and former) general population.

Example of conversion for age group 60-64: $103.8 \cdot 0.85 = 88.23$ smoking-related incidences per 100,000 people (never-smokers, current smokers and former smokers). $88.23/0.555 = 159.0$ smoking-related incidences per 100,000 smokers (current and former).

Age	Incidence per 100,000	Incidence in %
20-24	0.9	0.0009%
25-29	0.0	0.0000%
30-34	2.6	0.0026%
35-39	2.9	0.0029%
40-44	3.5	0.0035%
45-49	7.0	0.0070%
50-54	31.9	0.0319%
55-59	94.2	0.0942%
60-64	159.0	0.1590%
65-69	283.8	0.2838%
70-74	393.6	0.3936%
75-79	471.4	0.4714%

Table A3: Incidence rates of lung cancer by age in the female smoking (current and former) general population in Denmark from 2016 (12).

Secondary oesophageal cancer at age 80

ERR/Gy = $0.071 \cdot MOD$ (95% CI: 0.015 to 0.222) from nested case-control study on breast cancer patients by Journy et al. 2020 (15). MOD = mean oesophageal dose.

We assumed that second malignancy induction was independent of fractionation, and consequently no fractionation corrected doses were not used.

The incidence rate of oesophageal cancer in the general population was found in the NORDCAN database (12), see Table A4.

Age	Incidence per 100,000	Incidence in %
20-24	0.0	0.0000%
25-29	0.0	0.0000%
30-34	0.0	0.0000%
35-39	0.0	0.0000%
40-44	0.0	0.0000%
45-49	1.0	0.0010%
50-54	4.3	0.0043%
55-59	7.2	0.0072%
60-64	11.1	0.0111%
65-69	12.7	0.0127%
70-74	22.8	0.0228%
75-79	22.6	0.0226%

Table A4: Incidence rates of oesophageal cancer by age in the female general population in Denmark from 2016 (12).

Coronary artery event at age 80

EAR of radiation-induced coronary artery events by age 80 was modelled using table S13 in the supplementary materials of the population-based case-control study on breast cancer patients by Darby et al. 2013 (16). Coronary artery events were defined as myocardial infarction, coronary revascularization, death from ischemic or unstable angina. Bivariate linear interpolation was used to assess absolute risk values between the table entries.

α/β for coronary artery event: 2 Gy from Darby et al. 2013 (16).
Model variables: Biological equivalent fractionation-corrected to 25 fractions mean heart dose, patient age and no or ≥ 1 cardiac risk factor.

Cardiac risk factors included current smoking, BMI ≥ 30 and history of ischemic heart disease, diabetes or chronic obstructive pulmonary disease (16). All patients were in this study modelled twice: assuming no pre-existing cardiac risk factors and assuming pre-existing cardiac risk factors.

The table from Darby et al. provided EAR estimates by age 80 for mean heart doses up to 10 Gy and from age 40 years. Two patients had a fractionation corrected mean heart dose above 10 Gy (12.4 Gy and 13.3 Gy) and were modelled with mean heart doses of 10 Gy. A 33-year-old patient was modelled from 40 years.

References

1. DeCesaris CM, Rice SR, Bentzen SM, et al. Quantification of Acute Skin Toxicities in Patients With Breast Cancer Undergoing Adjuvant Proton versus Photon Radiation Therapy: A Single Institutional Experience. Int. J. Radiat. Oncol. Biol. Phys. 2019;104:1084–1090.

2. Marks LB, Bentzen SM, Deasy JO, et al. Radiation Dose–Volume Effects in the Lung. Int. J. Radiat. Oncol. 2010;76:S70–S76.

3. Bentzen SM, Dische S. Morbidity related to axillary irradiation in the treatment of breast cancer. Acta Oncol. 2000;39:337–347.

4. Belderbos J, Heemsbergen W, Hoogeman M, et al. Acute esophageal toxicity in non-small cell lung cancer patients after high dose conformal radiotherapy. Radiother. Oncol. 2005;75:157–164.

5. Tucker SL, Xu T, Paganetti H, et al. Validation of Effective Dose as a Better Predictor of Radiation Pneumonitis Risk Than Mean Lung Dose: Secondary Analysis of a Randomized Trial. Int. J. Radiat. Oncol. Biol. Phys. 2019;103:403–410.

6. Joiner MC, van der Kogel A. Basic Clinical Radiobiology, 4th ed, CRC Press; 2009.

7. Mukesh MB, Harris E, Collette S, et al. Normal tissue complication probability (NTCP) parameters for breast fibrosis: Pooled results from two randomised trials. Radiother. Oncol. 2013;108:293–298.

8. Huang H, Roberson J, Hou W, et al. NTCP model for hypothyroidism after supraclavicular-directed radiation therapy for breast cancer. Radiother. Oncol. 2021;154:87–92.

9. Brodin NP, Vogelius IR, Maraldo M V., et al. Life years lost-comparing potentially fatal late complications after radiotherapy for pediatric medulloblastoma on a common scale. Cancer. 2012;118:5432–5440.

10. Statistics Denmark. Survivors per 100,000 by age for females in Denmark from 2015 to 2019. Accessed: 2021, May 18. https://www.statistikbanken.dk.

11. Grantzau T, Thomsen MS, Væth M, et al. Risk of second primary lung cancer in women after radiotherapy for breast cancer. Radiother. Oncol. 2014;111:366–373.

12. NORDCAN. Cancer incidence per 100,000 by age for females in Denmark 2016. Accessed: 2021, May 18. https://www-dep.iarc.fr/nordcan/English/frame.asp.

13. Danish Health Authority. Smoking habits in Denmark 2018. Accessed: 2021, May 18. https://www.sst.dk/da/udgivelser/2019/danskernes-rygevaner-2018.

14. Centers for Disease Control and Prevention (CDC). What Are the Risk Factors for Lung Cancer? Accessed: 2021, May 18. https://www.cdc.gov/cancer/lung/basic_info/risk_factors.htm.

15. Journy N, Schonfeld SJ, Hauptmann M, et al. Dose-volume effects of breast cancer radiation therapy on the risk of second oesophageal cancer. Radiother. Oncol. 2020;151:33–39.

16. Darby SC, Ewertz M, McGale P, et al. Risk of ischemic heart disease in women after radiotherapy for breast cancer. N. Engl. J. Med. 2013;368:987–998.

Appendix B

	Photon [median (range)]	Proton [median (range)]	p
Early toxicities			
NTCP of radiation dermatitis	18.3% (5.4-41.7)	58.4% (31.4–69.7)	<0.001
NTCP of radiation pneumonitis	6.8% (3.3–13.6)	3.9% (2.7–9.1)	<0.001
NTCP of acute oesophageal toxicity*	3.3% (3.3–3.9)	3.6% (3.3–4.7)	0.11
Late, non-life-threatening toxicities			
NTCP of lung fibrosis	0.8% (0.0–4.0)	0.0% (0.0–1.3)	<0.001
NTCP of breast fibrosis	15.7% (11.0–18.0)	15.6% (11.2–16.3)	0.007
NTCP of hypothyroidism*	25.3% (3.9-49.1)	32.8% (3.2-56.3)	0.11
Late, life-threatening toxicities			
EAR of coronary artery event (no RF)	0.4% (0.0–3.2)	0.1% (0.0–0.7)	<0.001
EAR of coronary artery event (risk RF)	1.0% (0.0–5.6)	0.2% (0.0–1.3)	<0.001
EAR of secondary lung cancer (smoker)	4.8% (0.0–17.0)	2.7% (0.0–13.6)	<0.001
EAR of secondary oesophageal cancer*	0.05% (0.0–0.3)	0.1% (0.0–0.2)	0.58

Table B1: Estimated risks of radiation-induced toxicity for photon and proton therapy in 24 bilateral breast cancer patients. Paired, two-tailed Wilcoxon signed-rank test was used to compare photon and proton therapy plans. Abbreviations: NTCP, normal tissue complication probability; EAR, excess absolute risk; RF, risk factors. *Only evaluated for patients receiving loco-regional irradiation (12 patients).

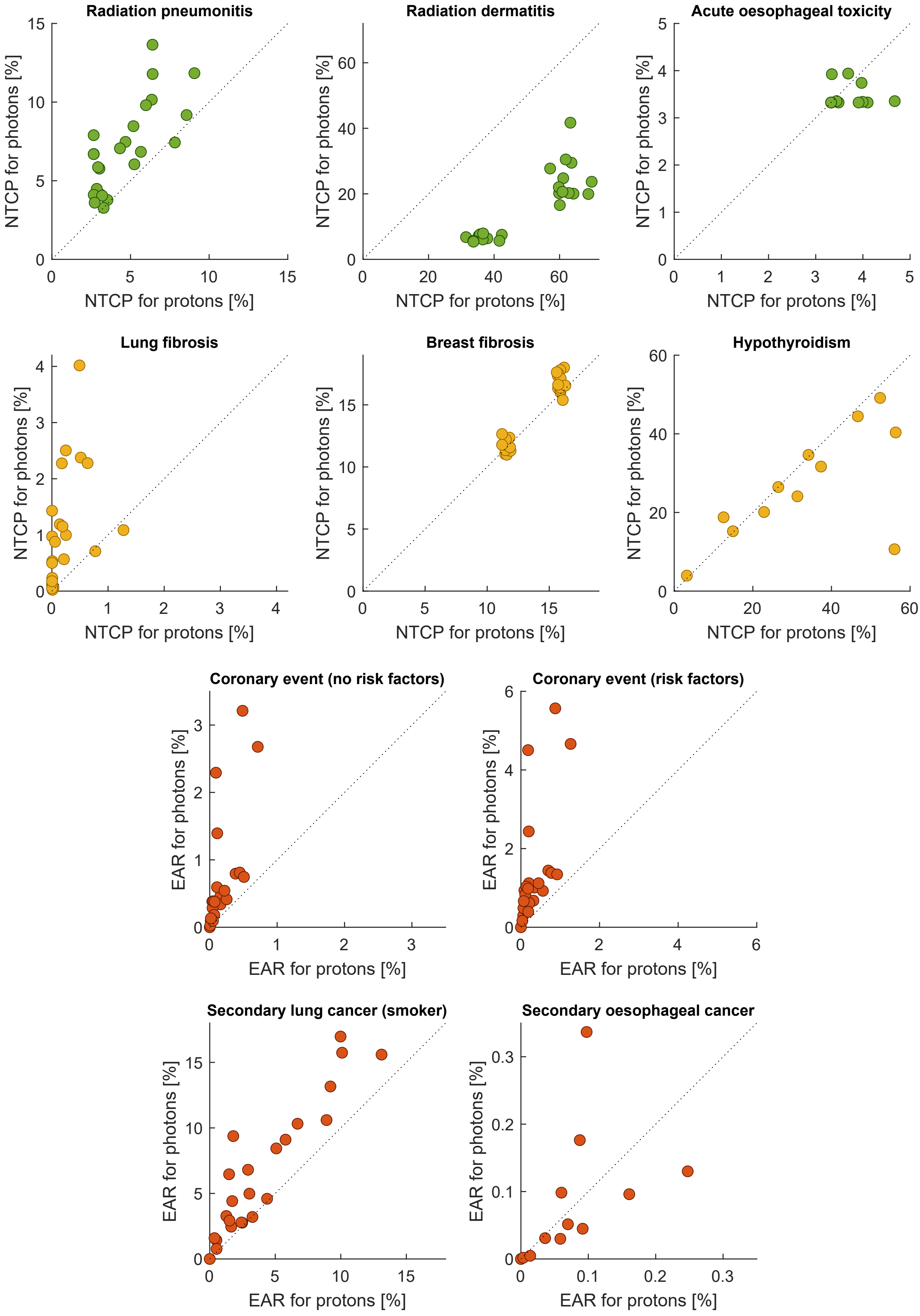

Figure B1: Normal tissue complication probability (NTCP) or excess absolute risk (EAR) at age 80 of the studied toxicities for the clinically delivered photon therapy plan and the comparative proton therapy plan. Risks of acute oesophageal toxicity, hypothyroidism and secondary oesophageal cancer were only evaluated in patients that received loco-regional irradiation. The toxicities were categorised in early (green), late, non-life-threatening (yellow) and late, life-threatening (red).
